# A Case of Paraplegia

**Published:** 1856-01

**Authors:** P. Van Patten

**Affiliations:** Walnut Camp, Ark.


### A CASE OF PARAPLEGIA.

BY P. VAN PATTEN, M. D., WALNUT CAMP, ARK.

Jan. 29th, 1855, called to see a negro girl, belonging to Miss St. C. Patient, æt. 9 years. Upon examination, found it to be a case of Paraplegia, supervening upon antero-posterior curvature of the spine. As far as could be determined by external exploration, the fifth, sixth, and seventh dorsal vertebræ were the only portions of the osseous structure principally involved; the transverse processes of the sixth were greatly enlarged, constituting a marked peculiarity. Entire inability to move the lower extremities, partial deprivation of sensation, with extreme placidity of the muscles present. Retention of urine and constipation had long been sources of distress and inconvenience. The forces of the organism were gradually, but constantly yielding: muco-purulent expectoration, with a harrassing cough, debility, &c., indicated too truly, how necessarily important lesions of the organic system affect the functions of animal life.

Upon inquiry, I learned that until three years previous, the girl had enjoyed good health, and had exhibited no indications of spinal curvature or disease,—that the first evidence of vetebral lesion appeared subsequent to having been run over by a wagon, the which passing over her back, transversely. Paralysis did not occur until nearly a year after the accident.

I proceeded at once to insert a seton on each side of the spinal column in the region of the sixth dorsal vertebræ. Directed the warm Soda bath, with smart friction to the extremities, to be used daily. Also exhibited

℞ Iodid. Potas. scru. j,
Iodin. Sublim. grs. v,
Aq. Pura ℥j,

S. Sixty drops twice a day—an hour before meals.

Likewise directed Ol. Terebinth, gtt. 10, once a day—to be given in mucilage. Diet to be broths, soft boiled eggs, &c., bed to be hard—mattrass preferable.

Feb. 3d. Called and found no appreciable change in regard to motion or sensation in parts affected, but was gratified to notice considerable diminution of cough; to learn that there was improvement of appetite and less urinary difficulty. Directed the use of Oint. Iodidii. with friction in the vicinity of the setons, once each day; all other treatment as before.

Continuing the above with modifications, according to circumstances, the use of tonics, &c., I had the satisfaction within six weeks, of discovering an increase of sensibility in the limbs, and witnessing an ability on the part of my patient of moving her toes. The setons were now repeated—the general and topical plan of treatment adhered to. In four months, the girl could walk and run about, deformity remaining. Discouraging as this case was, the result vindicated perseverance and assiduous attention, illustrating the fact that a rational attempt at success is preferable to entire doubt of amendment and supine do-nothingism.

*Remark.*—It is not always easy to diagnose between paralysis induced by pressure of the vertebræ upon the medulla spinalis, and that dependent upon inflammation and irritation. To distinguish accurately, is in a practical light important, as determining the question of treatment. If Mr. Percival Pott in treating on vertebral disease, is correct in the assertion that the palsy resulting from pressure is *invariably* attended with notable flaccidity and attenuation of the muscles of the extremities, and that paraplegia from inflammation and changes in the vertebræ and intervertebral substance is as constantly remarkable for the firmness and fullness of that part of the muscular system involved—then, our case presents in view of its origin, course and termination, peculiar features; firstly, because of curvature and paralysis with extreme softness and attenuation of the muscles, supervening so long after the mechanical injury received, viz: one year; secondly in view of its ready amenability to the treatment instituted.

*Querry.*—Can we reasonably conclude the interruption of innervation, as detailed, to have been produced by so grave and (usually) persistent cause as the displacement of osseous substance? I think not. *If then, we cannot admit the supposition, has Mr. Pott assumed too much in declaring firmness or flaccidity, as the case may be, invariably pathognomonic?*

Dec. 7th, 1855.

